# FGL2 deficiency alleviates maternal inflammation-induced blood-brain barrier damage by blocking PI3K/NF-κB mediated endothelial oxidative stress

**DOI:** 10.3389/fimmu.2023.1157027

**Published:** 2023-03-27

**Authors:** Lianjing Huang, Di Zhan, Ying Xing, Yaqin Yan, Qing Li, Jingyi Zhang, Sujuan Li, Qin Ning, Cai Zhang, Xiaoping Luo

**Affiliations:** ^1^ Department of Pediatrics, Tongji Hospital, Tongji Medical College, Huazhong University of Science and Technology, Wuhan, Hubei, China; ^2^ Department of Infectious Diseases, Tongji Hospital, Tongji Medical College, Huazhong University of Science and Technology, Wuhan, Hubei, China

**Keywords:** fibrinogen-like protein 2, maternal inflammation, blood-brain barrier, endothelial cells, oxidative stress

## Abstract

**Introduction:**

The impairment of blood-brain barrier (BBB) is one of the key contributors to maternal inflammation induced brain damage in offspring. Our previous studies showed Fibrinogen-like protein 2 (FGL2) deficiency alleviated maternal inflammation induced perinatal brain damage. However, its role in BBB remains undefined.

**Methods:**

Lipopolysaccharide (LPS) was intraperitoneally injected to dams at Embryonic day 17 to establish maternal inflammation model. FGL2 knockout mice and primary brain microvascular endothelial cells (BMECs) were used for the *in-vivo* and *in-vitro* experiments. BBB integrity was assessed by sodium fluorescein extravasation and tight junction (TJ) protein expression. Oxidative stress and the activation of PI3K/NF-κB pathway were evaluated to explore the mechanisms underlying.

**Results:**

Upon maternal inflammation, BBB integrity was remarkedly reduced in neonatal mice. Meanwhile, FGL2 expression was consistently increased in BBB-impaired brain as well as in LPS-treated BMECs. Moreover, FGL2 deficiency attenuated the hyperpermeability of BBB, prevented the decline of TJ proteins, and reduced the cytokine expressions in LPS-exposed pups. Mechanistically, the indicators of oxidative stress, as well as the activation of PI3K/NF-κB pathway, were upregulated after LPS exposure *in vivo* and *in vitro*. FGL2 deletion decreased the generation of ROS and NO, reduced the endothelial iNOS and NOX2 expressions, and suppressed the PI3K/NF-κB pathway activation. Besides, inhibition of PI3K by LY294002 decreased the oxidative stress in LPS-treated wild-type BMECs. While, overexpression of PI3K by lentivirus reemerged the induction of NOX2 and iNOS as well as NF-κB activation in FGL2-deleted BMECs.

**Conclusion:**

Our findings indicate that FGL2 deficiency alleviates the maternal inflammation-induced BBB disruption by inhibiting PI3K/NF-κB mediated oxidative stress in BMECs. Targeting FGL2 may provide a new therapy for prenatal brain damage of offspring.

## 1 Introduction

Fetal brain development is particularly susceptible to environmental influence. Increasing evidences have indicated that maternal inflammation during pregnancy negatively affects the organization and differentiation of fetal brain, leading to fetal brain injury, such as cerebral palsy (1–3). Moreover, prenatal brain damage by maternal inflammation is the major risk factor for later-onset neurodevelopmental disorders in offspring, such as autism spectrum disorder (4–6). Thus, prenatal period is the key window for manipulating the short-term and long-term outcomes of offspring. Using animal models that pregnant dams were intraperitoneally injected with lipopolysaccharide (LPS) or polyinosinic:polycytidylic acid (Poly(I:C)), related studies have made great progress in the behavioral phenotypes of adult offspring (5, 7). Whereas a lot is known about the influence of maternal inflammation on the developing brain, the understanding of underlying mechanisms is relatively less.

Blood-brain barrier (BBB) is a selective barrier that separates the brain from the circulation. The brain microvascular endothelial cells (BMECs) connected by tight junctions (TJs) are the core components of the BBB and primarily dictate the barrier properties of the BBB (8, 9). Normal functioning of the BBB protects the brain from inflammatory damage by restricting the infusion of pro-inflammatory mediators into the central nervous system (CNS) (10). BBB abnormality appears to be the common pathological factor of many maternal inflammation-related CNS diseases, including schizophrenia, epilepsy, and autism spectrum disorder (11–13). Recently, convincing study confirmed the causal effect of gestational maternal inflammation on long-lasting BBB dysfunction and lifelong brain inflammation in adult offspring (14). Moreover, the studies further indicated that BBB dysfunction in fetal brain occurred within a short time after maternal inflammation exposure (14, 15). Therefore, the prenatal impairment of BBB is increasingly recognized as the important etiological factor of maternal inflammation-derived neurodevelopmental disorders (14, 15). However, the studies of BBB dysfunction in the developing brain are still limited, and the dynamic changes of BBB during maternal inflammation along with the underlying mechanisms still lag behind.

Fibrinogen-like protein 2 (FGL2) is a member of the fibrinogen-related protein superfamily, which is comprised of soluble FGL2 (sFGL2) and membrane-bound FGL2 (mFGL2) (16). sFGL2 is predominantly generated by T cells, possessing an immunosuppressive effect (16). While, mFGL2 is constitutively expressed on normal macrophages and endothelial cells and induced by cytokines or viruses, playing as an inflammatory procoagulant protein (16–18). Our previous studies have indicated the importance of mFGL2 in maternal inflammation-induced white matter injury and neuroinflammation of offspring, and the regulation of mFGL2 on microglial phenotypes is involved in this process (19). However, we noticed that the protection of FGL2 deficiency on neuroinflammation had already existed before the transformation of microglial phenotypes (19). Glial activation and BBB impairment are the key events mediating neuroinflammation (9). However, their interaction and contribution to neuroinflammation during maternal inflammation remained unclear. BBB dysfunction has been reported as an early feature of many FGL2-related neuroinflammatory diseases, including traumatic brain injury and intracerebral hemorrhage (20, 21). More importantly, BBB disruption in these diseases precedes neuroinflammatory responses identified by glia activation (9). It implied that in addition to microglia, FGL2 might also participate in early regulatory stage of inflammatory response, such as BBB function. Since endothelial cells are the core component of BBB and possess high expressions of FGL2 under cytokine stimulation, we speculate that FGL2 may have an essential impact on BBB integrity in the neonatal brain after maternal inflammation exposure. In the present study, we explored the regulatory effect and mechanism of FGL2, especially endothelium-expressed FGL2, on neonatal BBB damage through the LPS-induced animal model, FGL2 knockout mice, and primary BMECs.

## 2 Materials and methods

### 2.1 Animals

Eight-week-old C57BL/6J wild-type (WT) mice were obtained from GemPharmatech Co., Ltd (Jiangsu, China). FGL2 knockout (FGL2^-/-^) C57BL/6J mice were constructed by Shanghai Model Organisms Center, Inc. (Shanghai, China) *via* CRISPR/Cas9 technology targeting the first and second exons of the mouse *FGL2* gene (17). Heterozygous mice (FGL2^+/-^) were generated by crossing FGL2^-/-^ mice and their wild-type littermates. The mice were identified by PCR genotyping of genomic DNA from toe biopsies. Genotyping of mice was performed with two pairs of primers: P1/P2, F 5’-AATGCGCCCGCCCTTTTCTGG-3’ and R 5’-CTGCCGCGGGAGCTGGATGGT-3’, resulting in a 302bp product; P3/P4, F 5’-TATGGTATCTTTTGGGCACTGGTA-3’ and R 5’-TTCTTGGGCCTAATCATCATCTTG-3’, resulting in a 468bp product. The expressions of FGL2 in the brain of WT and FGL2^-/-^ mice were confirmed by RT-qPCR and western blotting. All mice were housed in the SPF animal experiment center of Tongji Hospital and pregnant mice were housed separately per cage. All the animal procedures were in accordance with Chinese guidelines and regulations for experimental animals, and approved by the Tongji Hospital Animal Care and Use Committee (number: TJH-201901018).

### 2.2 Maternal inflammation animal model

At Embryonic day (E) 17, pregnant dams were weighted and randomly intraperitoneally injected with 200 μg/kg lipopolysaccharide (*Escherichia coli*, serotype 0111: B4, L2630; Sigma-Aldrich, St. Louis, MO, USA) or 100 μl phosphate buffer saline (PBS) in accordance with the previous studies (19, 22). Dams were sacrificed at E18 or allowed to deliver at term (E19-20), while pups were sacrificed one or seven days after birth. The placenta was washed in PBS solution to remove the maternal blood in tissue, and the brain was rolled carefully on dried filter paper to clean the surface.

### 2.3 Measurement of BBB permeability

The BBB permeability was assessed as described previously using sodium fluorescein (NaF) with slight modifications (23). All the operations below were avoided from light. 5 μl of 0.1 g/ml NaF (Solarbio, Beijing, China) was injected intraperitoneally. One hour after injection, brain tissues and serum were collected. Brain tissues were weighted and homogenized in cold-PBS and centrifugated at 14000 g for 5 min at 4°C. The supernatants were mixed with an equal volume of 15% trichloroacetic acid followed by centrifugation at 1000 g for 10 min. Serum was also mixed with ten times the serum volume of 15% trichloroacetic acid before centrifugation. 25 μl of 5 M NaOH were added in 100 μl of the reaction solutions above before measurement. The fluorescence was measured (excitation 485/20 nm, emission 528/20 nm; Synergy HT, BioTek, Vermont, USA), and the fluorescence intensity was calculated (the fluorescence intensity = brain fluorescence/serum fluorescence).

### 2.4 Immunofluorescence staining

According to our previous study, brain tissues were fixed in 4% paraformaldehyde, dehydrated by gradient ethanol, embedded in paraffin, and sectioned at 6 μm (19). The slides were then deparaffinized with xylene, rehydrated through graded ethanol, antigen retrieved by boiled citrate buffer under high temperature and high pressure for 2 min, and blocked in 5% goat serum (19). The cell slides were fixed in 4% paraformaldehyde directly and blocked in 5% goat serum. After being blocked, the slides were incubated with primary antibodies overnight at 4°C. After being incubated with the appropriate secondary antibodies (1:200, Life technologies, California, USA) at room temperature for 2 h, the sections were visualized and photographed using a fluorescence microscope (IX71, OLYMPUS, Tokyo, Japan). DAPI (Servicebio, Wuhan, China) was used for nuclear staining. The primary antibodies used were as follows: FGL2 (1:500, Abnova Cat# H00010875-M01), NOX2 (1:100, Proteintech Cat# 19013-1-AP), iNOS (1:100, Proteintech Cat# 18985-1-AP), vWF (1:100, Abcam Cat#ab11713), CD31 (1:2000, Abcam Cat# ab182981; 1:400, Abcam Cat# ab56299). For quantification, the fluorescence intensity or the percentage of at least 3 neonatal brains in each group was analyzed by the ImageJ software, and 3-5 different visual fields per animal were chosen for analysis. The fluorescence intensity of FGL2 or iNOS in CD31+ vessels was analyzed through selecting the CD31+ vessels as our region of interest (ROI) followed by “% Area” function of ImageJ in the ROI of each section. Meanwhile, the number of CD31+ and NOX2+/CD31+ cells in the cortex was counted manually using the “Multi-point” tool of Image J and the percentage of NOX2+ cells in CD31+ cells was calculated according to the reference (24).

### 2.5 Isolation and culture of primary brain microvascular endothelial cells

Primary brain microvascular endothelial cells (BMECs) were prepared from 10-day-old C57BL/6J mice according to Liu’s protocol with minimal modifications (25). Briefly, the gray matters were isolated from the brain and minced in cold D-Hanks’ solution. The homogenate was supplied with 25% BSA and centrifugated at 600 g for 10 min at 4°C. The deposition in the lowest layer was collected. After three times of centrifugation, all microvessel pellets were further digested with 0.1% collage type II (Sigma-Aldrich, St. Louis, MO, USA) for 20 min at 37°C and finally cultured in DMEM/F12 medium supplemented with 20% FBS, 100 U/ml penicillin-streptomycin at 37°C, 5% CO_2_. Culture medium was changed the next day and renewed every 2–3 days. When the cells reached 90% confluency, they were washed with D-hanks’ solution and digested by trypsin-EDTA for 3–4 min at 37°C until the majority of long spindle-shape cells contracted to round. After being suspended with fresh full medium, the cells were seeded into culture flasks at an appropriate density. The characterizations of BMECs were observed by morphology and immunofluorescent staining.

### 2.6 Measurement of ROS in brain and in BMECs

The levels of reactive oxygen species (ROS) in brain or cultured cells were assessed by dihydroethidium (DHE; Beyotime, Shanghai, China), according to manufacturers’ instructions and the previous study (26). All the operations below were avoided from light. Fresh brain tissues were sectioned to frozen sections at 6 μm, incubated with DHE for 40 min at 37°C, and directly observed using a fluorescence microscope (IX71, OLYMPUS, Tokyo, Japan) after nuclear staining, further quantification was used “% Area” function of the ImageJ software and 3-5 different visual fields per animal were chosen for analysis. BMECs were seeded in 12-well plates or 96-well black plates. After treatment, living cells were incubated directly with DHE for 30 min. The fluorescence intensity was observed in 12-well plates using the fluorescence microscope (IX71, OLYMPUS, Tokyo, Japan) or measured in 96-well plates by a fluorescence spectrophotometer (excitation 360/40 nm, emission 460/40 nm; Synergy HT, BioTek, Vermont, USA).

### 2.7 Measurement of NO content in brain and in BMECs

The concentrations of NO in brain or culture supernatant were assessed by Griess reagent in the Total Nitric Oxide Assay Kit (Beyotime, Shanghai, China) according to manufacturers’ instructions and the previous study (27). The absorbance was measured at 540 nm using a microplate spectrophotometer (Synergy HT, BioTek, Vermont, USA), and NO levels were calculated using a standard curve prepared by standard nitrite solutions.

### 2.8 Adenovirus and lentivirus infection

To suppress the FGL2 expression, the plasmid containing double-stranded miRNA templates for FGL2 (NM_008013) was constructed into adenovirus vector (pADM-CMV-GFP) by Vigene Biosciences Co., Ltd. (Shandong, China). The oligonucleotides encoding FGL2-miRNA sequences were the same as the previous study (28). BMECs were infected at a multiplicity of infection (MOI) of 250 and supplied with 5 μg/ml polybrene. To overexpress the PI3K expression, the flag-tagged PI3K overexpressed plasmid (NM_001077495) was constructed into lentivirus vector (pHBLV-CMV-MCS-ZsGreen-T2A-PURO) by Hanbio Biotechnology Co., Ltd. (Shanghai, China). BMECs were infected at a MOI of 40 and supplied with 5 μg/ml polybrene. 48 h after infection, the fluorescence intensity of cells was observed under a fluorescence microscope (IX71, OLYMPUS, Tokyo, Japan). The expressions of FGL2 and flag were detected by western blotting.

### 2.9 Western blotting

Proteins from tissues and cells were extracted using RIPA lysis buffer (BOSTER, Wuhan, China) and quantified by BCA Protein Assay kit (BOSTER, Wuhan, China). The samples were then separated by 10% SDS/PAGE gel and transferred onto nitrocellulose membranes. The membranes were subsequently blocked with 5% skim milk for 1h at room temperature and incubated with the primary antibodies (**Table S1**) overnight at 4°C. The next day, the membranes were incubated with HRP-conjugated secondary antibodies (1:5000, Servicebio, Wuhan, China) for 1 h at 37°C. The images were further detected with an enhanced chemiluminescence system (ChemiDoc XRS+, Bio-Rad, California, USA) and analyzed using ImageLab software. GAPDH or ACTIN was used to normalize the expressions of protein.

### 2.10 Quantitative real-time PCR

Total RNA from tissues and cells was extracted with RNAiso Plus (Takara, Kyoto, Japan). The concentration and quality of RNA were detected with a NanoDrop (Thermo Fisher Scientific, Waltham, USA). cDNA was prepared using PrimeScript RT Master Mix Kit (Takara, Kyoto, Japan). Quantitative real-time PCR was performed on CFX Connect Real-Time System (Bio-Rad, California, USA) using TB Green Premix EX Taq (Takara, Kyoto, Japan). The data was analyzed by 2^-ΔΔCt^ and normalized to *GAPDH*. The primer sequences were listed in **Table S2**.

### 2.11 Statistical analysis

All statistical analyses were performed using GraphPad Prism^®^ 8.0 (GraphPad Software, Inc., California, USA) and date were expressed as the means ± standard error of the mean (SEM). Unpaired two-tailed Student’s *t* test was used for comparisons between two groups and one-way analysis of variance (ANOVA) with Tukey’s *post-hoc* test was for multiple groups. *P* value of <0.05 was considered statistically significant. The number of animals or samples in each group was indicated in the figure legends and the data was the representative of at least three independent experiments.

## 3 Results

### 3.1 Increased FGL2 expression was associated with reduced tight junctions in LPS-exposed neonatal mice

**Figure 1:**
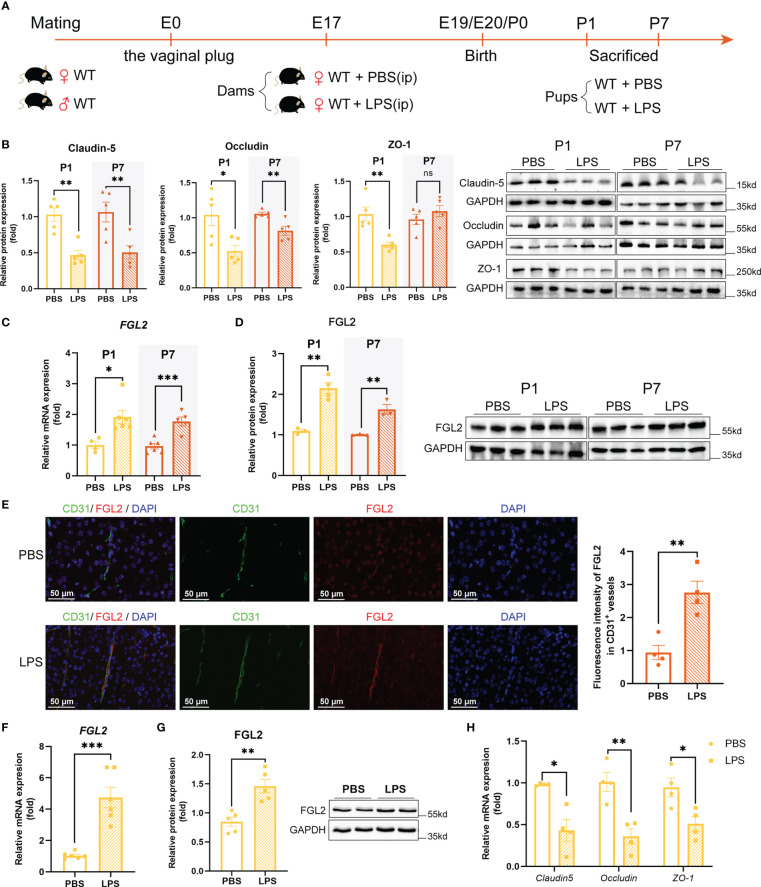
Increased endothelial expression of FGL2 was associated with the reduced TJ proteins. **(A)** Experimental scheme of WT mice. WT dams were randomly intraperitoneally injected with LPS (200 µg/kg) or PBS at E17. Pups were sacrificed and their brains were collected at P1 and P7. **(B)** The protein expressions of Claudin-5, Occludin, and ZO-1 in brain at P1 and in cortex at P7 (*n* = 5). **(C)** The mRNA levels of FGL2 in brain at P1 and in cortex at P7 (*n* = 4:6:6:5). **(D)** The protein expressions of FGL2 in brain at P1 and in cortex at P7 (*n* = 3:4:3:3). **(E)** FGL2 expression in CD31+ vessels by immunofluorescence staining in cortex at P7. The fluorescence intensity of FGL2 in CD31+ vessels was analyzed by the ImageJ software and normalized to controls (*n* = 4). Representative images and quantitative results were shown. Scale bar=50 μm. **(F)** The mRNA levels of FGL2 in BMECs (*n* = 6). BMECs were treated with LPS (5μg/ml) or PBS for 24h. **(G)** The protein expressions of FGL2 in BMECs (*n* = 5). **(H)** The mRNA levels of Claudin-5, Occludin, and ZO-1 in BMECs (*n* = 4). The data was expressed as mean ± SEM and was the representative of at least three independent experiments. Statistical comparisons were assessed by unpaired two-tailed Student’s *t* test. ns *P*>0.05, *P<0.05, **P<0.01, ***P<0.001.

Wild-type dams were intraperitoneally injected with 200 µg/kg LPS or PBS at E17. The schematic of experimental design was shown in **Figure 1A**. 24h after injection, the expressions of pro-inflammatory cytokines in the placenta were all significantly increased, including TNF-α, IL-1β, and IL-6, while the anti-inflammatory IL-10 expression was decreased (**Figure S1A**). It suggested the reliable construction of maternal inflammation mouse model. At the dose used, LPS decreased the maternal weight 24h post injection but had no significant effect on time of birth and litter size compared with controls (**Figures S1B–D**). Occludin, Claudin-5, and ZO-1 were the representative tight junction (TJ) proteins, performing a crucial role in maintaining BBB integrity (29). Therefore, the expressions of TJ proteins in the brain of postnatal pups were further assessed. Compared with controls, LPS-exposed pups showed significantly decreased cerebral expressions of Claudin-5, Occludin, and ZO-1 at postnatal day 1 (P1) (**Figure 1B**). Additionally, the expressions of Claudin-5 and Occludin were still lower than controls at postnatal day 7 (P7) (**Figure 1B**). These results indicated the tight junctions were reduced in the brain of neonatal mice with maternal inflammation, implying the impaired BBB integrity.

To explore the role of FGL2 in BBB integrity under the condition of maternal inflammation, we firstly analyzed the expression of FGL2 in LPS-exposed pups. Compared with controls, cerebral mRNA and protein expressions of FGL2 were remarkably increased in LPS-exposed pups at both P1 and P7 (**Figures 1C, D**). Moreover, FGL2 expression in cerebral vessels was precisely investigated by immunofluorescence staining. It showed that the fluorescence intensity of FGL2 in CD31-positive vessels was also significantly elevated in LPS-exposed pups (**Figure 1E**). We then took advantage of LPS-treated primary BMECs to further confirm the link between endothelial FGL2 and TJ proteins. As previously (25), primary BMECs presented a swirl monolayer and expressed von Willebrand factor (vWF), an endothelial specific marker (**Figure S2**). Moreover, the results showed that the mRNA and protein expressions of FGL2 in LPS-treated BMECs were markedly increased (**Figures 1F, G**), while the mRNA levels of TJ proteins, including Claudin-5, Occludin, and ZO-1, were significantly decreased (**Figure 1H**). These data strongly suggested that the increased endothelial expression of FGL2 in vessels was associated with the BBB integrity in maternal inflammation-exposed neonatal mice.

### 3.2 FGL2 deficiency attenuated BBB damage and brain inflammation in LPS-exposed neonatal mice

**Figure 2:**
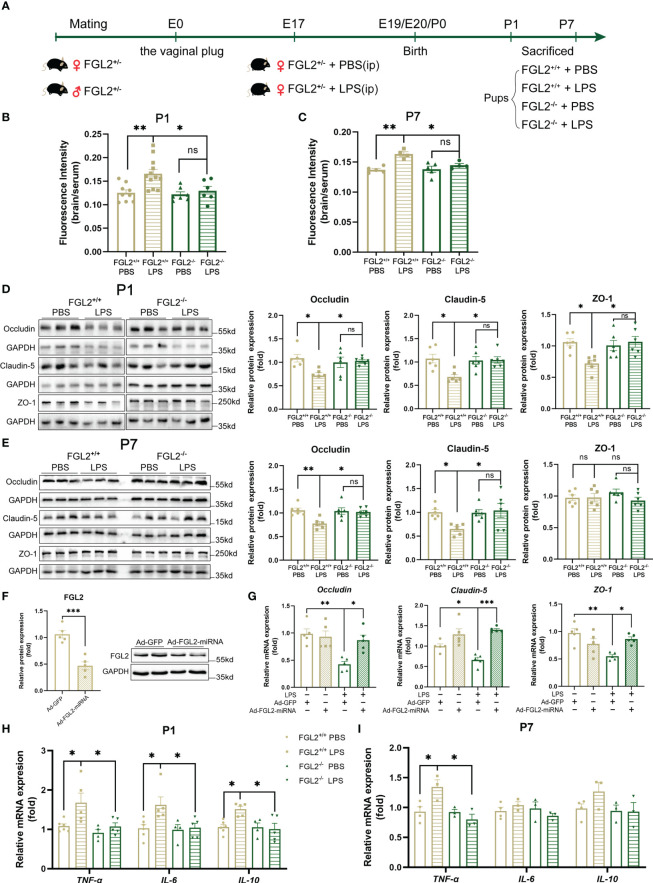
FGL2 deficiency attenuated maternal inflammation-induced BBB damage and neuroinflammation. **(A)** Experimental scheme of FGL2^+/-^ mice. FGL2^+/-^ dams were randomly injected with LPS (200 µg/kg) or PBS at E17. FGL2^+/+^ and FGL2^-/-^ pups were sacrificed and their brains were collected at P1 and P7. **(B)** The permeability of BBB measured by NaF in pups at P1 (*n* = 9:12:7:6). **(C)** The permeability of BBB measured by NaF in pups at P7 (*n* = 5:4:5:4). **(D)** The protein expressions of Occludin, Claudin-5, and ZO-1 in brain at P1 (*n* = 6). **(E)** The protein expressions of Occludin, Claudin-5, and ZO-1 in cortex at P7 (*n* = 6). **(F)** The protein expression of FGL2 in BMECs (*n* = 5). BMECs were infected by Ad-FGL2-miRNA or Ad-GFP for 48h. **(G)** The mRNA levels of *Claudin-5*, *Occludin*, and *ZO-1* in BMECs (*n* = 5). BMECs were infected by adenovirus for 48 h before treatment of LPS (5 μg/ml) or PBS for 24h. **(H)** The mRNA levels of *TNF-α*, *IL-6*, and *IL-10* in brain at P1 (*n* = 6:5:4:5). **(I)** The mRNA levels of *TNF-α*, *IL-6*, and *IL-10* in cortex at P7 (*n* = 4:3:3:3). The data was expressed as mean ± SEM and was the representative of at least three independent experiments. Statistical differences were assessed by unpaired two-tailed Student’s t test for two groups and one-way ANOVA with Tukey’s *post-hoc* test for four groups. ns *P*>0.05, *P<0.05, **P<0.01, ***P<0.001.

To further investigate the role of FGL2 in BBB function, we established FGL2^-/-^ mice and confirmed the cerebral knockout of FGL2 by RT-qPCR and western blotting (**Figure S3**). In order to exclude the influence of maternal FGL2 levels on the offspring, we treated FGL2^+/-^ dams with LPS and explored the effect of FGL2 on FGL2^+/+^ pups and their FGL2^-/-^ littermates (**Figure 2A**). Compared with controls, the reductions in both body weight and brain weight were found in LPS-exposed FGL2^+/+^ pups at P1 (**Figures S4A–D**), while FGL2^-/-^ littermates were resistant to the LPS-induced loss of brain weight at P1 (**Figure S4B**). However, there was no significant difference in brain-to-body weight ratio among the four groups at both P1 and P7 (**Figures S4E, F**). Moreover, we found the induction of pro-inflammatory cytokines in the placenta from FGL2^+/-^ dams after LPS treatment (**Figure S5A**), as well as the increased cerebral FGL2 expression in their FGL2^+/+^ pups (**Figures S5B, C**).

Next, we compared the BBB permeability in FGL2^+/+^ and FGL2^-/-^ neonatal mice. After LPS exposure, the relative fluorescence intensities of NaF in the brain of FGL2^+/+^ pups were obviously higher than PBS-treated controls at both P1 and P7 (**Figures 2B, C**), suggesting the hyperpermeability of BBB in conditions of maternal inflammation. In contrast with FGL2^+/+^ pups, the hyperpermeability of BBB was notably attenuated in FGL2^-/-^ littermates at both P1 and P7, indicated by the reduced NaF fluorescence intensity in the brain (**Figures 2B, C**). These results pointed out that FGL2 depletion restored the BBB permeability during maternal inflammation. Tight junctions are the important components determining the BBB permeability (30). Consistent with the increased permeability, the expressions of TJ proteins were significantly reduced in LPS-treated FGL2^+/+^ pups (**Figures 2D, E**). However, in FGL2^-/-^ pups the LPS-induced reduction of TJ proteins was significantly mitigated (**Figures 2D, E**). Moreover, when FGL2 expression was suppressed by adenovirus *in vitro* (**Figure 2F**), we also found that the reduction of mRNA expressions of *Claudin-5*, *Occludin*, and *ZO-1* were all alleviated in LPS-treated BMECs (**Figure 2G**). These data highly suggested FGL2 deficiency restored the function of BBB by upregulating the expressions of TJ proteins and recovering the permeability of BBB in maternal inflammation-exposed pups.

Furthermore, we evaluated the mRNA levels of cytokines in brains of FGL2^+/+^ and FGL2^-/-^ offspring. Consistent with our previous study in BALB/c mice (19), we observed elevated cytokines, including TNF-α, IL-6, and IL-10, in LPS-exposed FGL2^+/+^ pups at P1 (**Figure 2H**). However, the eruption of pro-inflammatory cytokines was strongly inhibited in FGL2^-/-^ littermates at P1 (**Figure 2H**), and the protection against inflammation was still significant on TNF-α at P7 (**Figure 2I**). Therefore, FGL2 deficiency showed the remarkable protection on neonatal BBB disruption, which might further decrease the levels of pro-inflammatory cytokines in the brain.

### 3.3 FGL2 deficiency protected against the maternal inflammation-induced oxidative stress in BMECs

**Figure 3:**
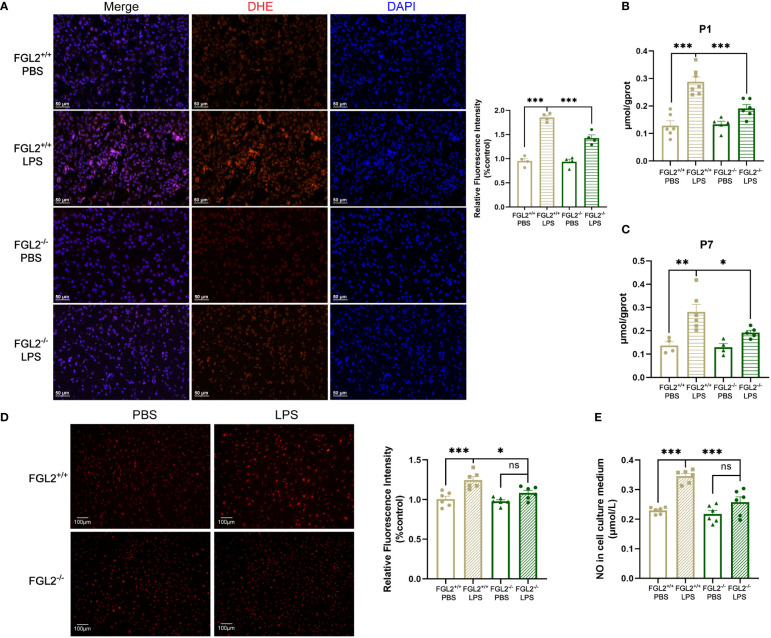
FGL2 deficiency decreased the maternal inflammation-induced generation of ROS and NO. **(A)** The level of ROS by DHE staining in cortex at P7 (*n* = 4). Representative images were shown and quantitative results were normalized to controls. Scale bar=50 μm. **(B)** The NO contents in brain at P1 (*n* = 6:7:5:6). **(C)** The NO contents in cortex at P7 (*n* = 4:6:4:5). **(D)** The level of ROS by DHE staining in BMECs (*n* = 6). FGL2^+/+^ and FGL2^-/-^ BMECs were treated with LPS (5 μg/ml) or PBS for 24 h. Representative images were shown, and quantitative results were normalized to controls. Scale bar=100 µm. **(E)** The concentration of NO in culture mediums of BMECs (*n* = 6). The data was expressed as mean ± SEM and was the representative of at least three independent experiments. Statistical differences were assessed by one-way ANOVA with Tukey’s *post-hoc* test for multiple groups. ns *P*>0.05, *P<0.05, **P<0.01, ***P<0.001.

Oxidative stress, due to uncontrolled accumulation of intracellular reactive oxygen species (ROS) and nitrogen species, is regarded as a crucial mechanism of BBB damage (9, 31). Previous study has observed that the imbalance of oxidation and antioxidant was concomitant with the BBB disruption in LPS-exposed fetal brain (15). To clarify the mechanisms of FGL2-mediated BBB damage, we further investigated oxidative stress in the brain of FGL2^+/+^ and FGL2^-/-^ pups. We found LPS-exposed FGL2^+/+^ brain exhibited a general increase in ROS levels *ex vivo*, indicated by the increased fluorescence intensity of DHE staining at P7 (**Figure 3A**). The contents of cerebral nitric oxide (NO) were also increased at P1 and P7 (**Figures 3B, C**). In contrast, FGL2 deficiency strikingly suppressed the inductions of ROS and NO (**Figures 3A–C**), suggesting the beneficial effects of FGL2 depletion on maternal inflammation-induced cerebral oxidative stress. Primary BMECs isolated from FGL2^+/+^ and FGL2^-/-^ mice were subsequently used to evaluate the endothelial production of ROS and NO. Similarly, the production of LPS-induced ROS and NO were markedly increased in FGL2^+/+^ BMECs but significantly inhibited in FGL2^-/-^ cells (**Figures 3D, E**).

**Figure 4:**
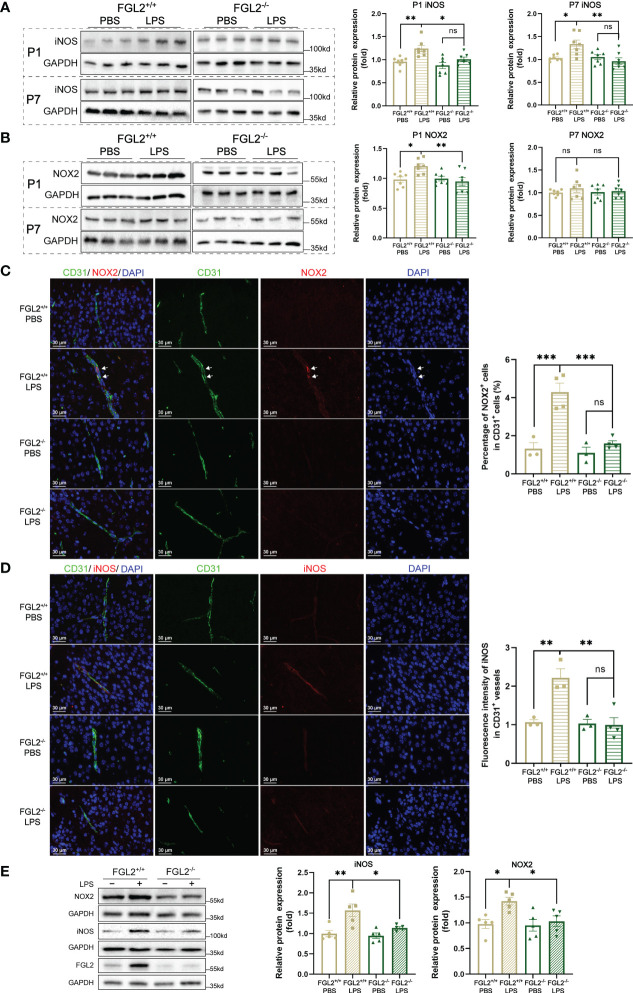
FGL2 deficiency decreased the maternal inflammation-induced endothelial expressions of NOX2 and iNOS. **(A)** The protein expressions of iNOS in brain at P1 and in cortex at P7 (*n* = 7). **(B)** The protein expressions of NOX2 in brain at P1 and in cortex at P7 (*n* = 7). **(C)** NOX2 expressions in CD31+ vessels by immunofluorescence staining in cortex at P7 (*n* = 3:4:3:4). The number of NOX2+ and CD31+ cells were counted manually by the ImageJ software, and the percentage was calculated. Representative images and quantitative results were shown. White arrows points to the representative cells. Scale bar= 30 μm. **(D)** iNOS expressions in CD31+ vessels by immunofluorescence staining in cortex at P7 (*n* = 3:3:3:4). The fluorescence intensity of iNOS in CD31+ vessels was analyzed by the ImageJ software and normalized to controls. Representative images and quantitative results were shown. Scale bar= 30 μm. **(E)** The protein expressions of iNOS and NOX2 in BMECs (*n* = 5). FGL2^+/+^ and FGL2^-/-^ BMECs were treated with LPS (5 μg/ml) or PBS for 24h. The data was expressed as mean ± SEM and was the representative of at least three independent experiments. Statistical differences were assessed by one-way ANOVA with Tukey’s *post-hoc* test for multiple groups. ns *P*>0.05, *P<0.05, **P<0.01, ***P<0.001.

In the context of BBB impairment, NADPH oxidase 2 (NOX2) and inducible nitric oxide synthase (iNOS) are responsible for the major production of ROS and NO, respectively (32–34). We further explored the regulation of FGL2 on NOX2 and iNOS expressions. The cerebral expressions of iNOS in pups were induced by LPS exposure and markedly inhibited upon FGL2 deficiency at both P1 and P7 (**Figure 4A**). Meanwhile, we observed the induction of NOX2 expression in LPS-exposed pups at P1, which was also inhibited by FGL2 deletion (**Figure 4B**). To precisely investigate the expressions of iNOS and NOX2 *in vivo*, we used immunofluorescence staining to analyze their vascular expressions in cortex at P7. After LPS exposure, the percentage of NOX2-positive cells in CD31-positive vessels was increased in FGL2^+/+^ pups and was obviously decreased in FGL2^-/-^ littermates (**Figure 4C**). Simultaneously, the fluorescence intensity of iNOS in CD31-positive vessels was also elevated by LPS and suppressed by FGL2 deletion (**Figure 4D**). Likewise, the endothelial expressions of NOX2 and iNOS were both increased in LPS-treated FGL2^+/+^ BMECs (**Figure 4E**). FGL2 deletion evidently downregulated the expressions of both proteins *in vitro* (**Figure 4E**). Together, these data pointed out that FGL2 deficiency dramatically decreased the vascular endothelial oxidative stress in the brain of neonatal mice upon maternal inflammation exposure.

### 3.4 FGL2 regulated the oxidative stress of BMECs *via* PI3K/NF-κB pathway

**Figure 5:**
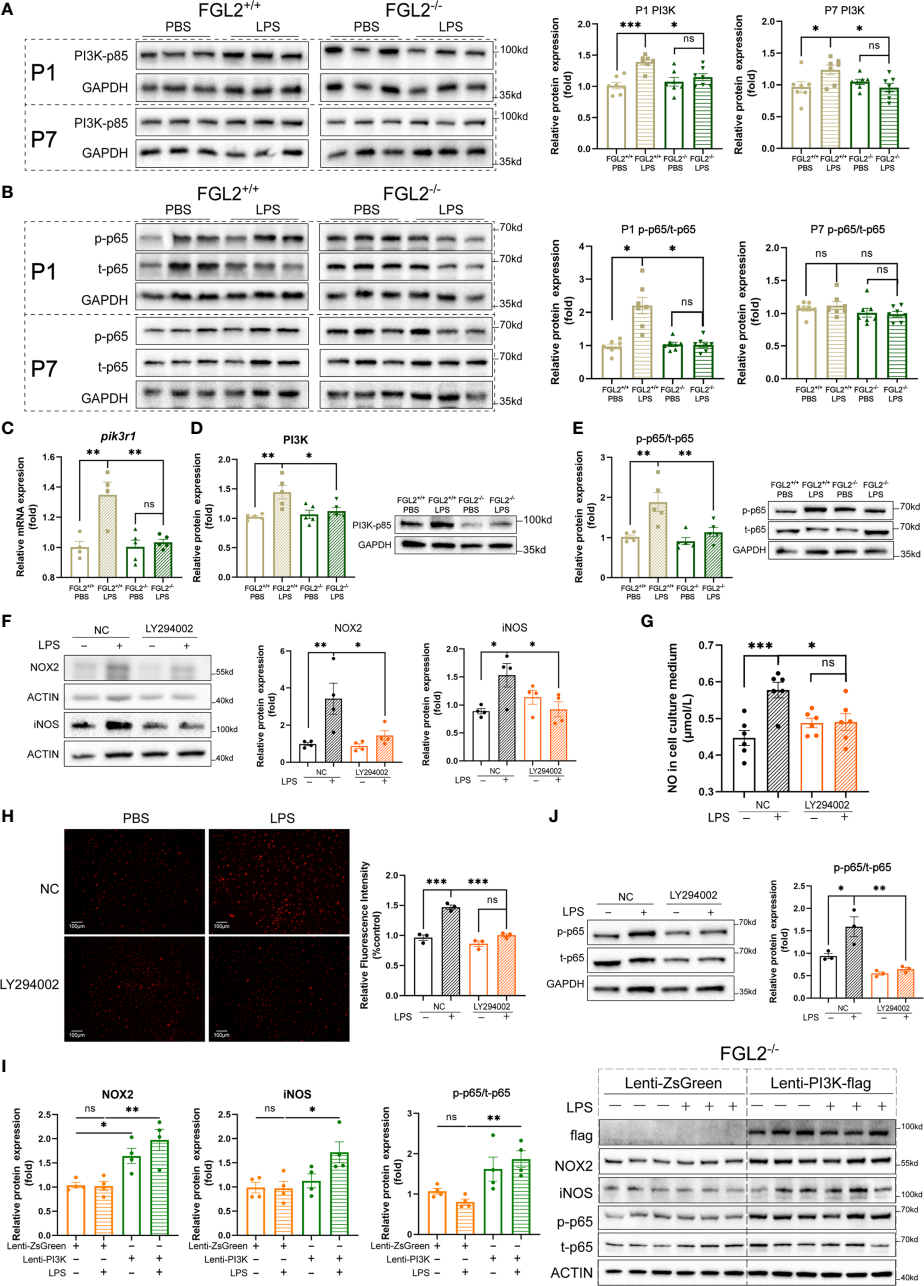
FGL2 deficiency alleviated endothelial oxidative stress *via* inhibiting the activation of PI3K/NF-κB pathway. **(A)** The protein expressions of PI3K-p85 in brain at P1 and in cortex at P7 (*n* = 7). **(B)** The protein expressions of NF-κB p65 and its phosphorylated form in brain at P1 and in cortex at P7 (*n* = 7). **(C)** The mRNA level of *pik3r1* in BMECs (*n* =4:4:5:5). FGL2^+/+^ and FGL2^-/-^ BMECs were treated with LPS (5 μg/ml) or PBS for 24 h. **(D)** The protein expression of PI3K-p85 in BMECs (*n* = 5). **(E)** The protein expressions of NF-κB p65 and its phosphorylated form in BMECs (*n* = 5). **(F)** The protein levels of NOX2 and iNOS in LY294002-pretreated BMECs (*n* = 4). Before LPS (5 μg/ml) stimulation for 24 h, FGL2^+/+^ BMECs were pretreated with or without LY294002 (20 μM) for 2 h. **(G)** The concentration of NO in culture mediums of LY294002-pretreated BMECs (*n* = 6). **(H)** The levels of ROS by DHE staining in LY294002-pretreated BMECs (*n* = 3). Representative images were shown, and quantitative results were normalized to controls. Scale bar=100 µm. **(I)** The protein expressions of flag, NOX2, iNOS, p-p65, and t-p65 in FGL2^-/-^ BMECs (*n* = 4). FGL2^-/-^ BMECs were infected by Lenti-PI3K-flag or Lenti-ZsGreen for 48 h before treatment of LPS (5 μg/ml) for 24 h. **(J)** The protein levels of p-p65 and t-p65 in LY294002-pretreated BMECs (*n* = 3). The data was expressed as mean ± SEM and was the representative of at least three independent experiments. Statistical differences were assessed by unpaired two-tailed Student’s t test for two groups and one-way ANOVA with Tukey’s *post-hoc* test for four groups. Welch’s ANOVA with Dunnett’s T3 *post-hoc* test was used to compare the protein expression of p-p65/t-p65 among four groups in brain at P1. ns *P*>0.05, *P<0.05, **P<0.01, ***P<0.001.

Previous studies have revealed that phosphatidylinositol 3-kinase (PI3K)-dependent pathway mediates the FGL2-induced chemokine production (35). More importantly, the regulatory subunit of PI3K, PI3K-p85, has been reported to regulate endothelial oxidative stress (36). To explore the molecular mechanisms underlying FGL2 facilitated endothelial oxidative stress, we detected the expression of PI3K-p85 in pups upon maternal inflammation exposure. Compared with controls, the cerebral expression of PI3K-p85 was elevated in LPS-exposed FGL2^+/+^ pups at both P1 and P7 (**Figure 5A**). In contrast, the increase of PI3K-p85 was significantly suppressed in FGL2^-/-^ littermates (**Figure 5A**). Nuclear factor kappa B (NF-κB) is an important downstream of PI3K and has been widely reported to be involved in oxidative stress (32). We found the activation of NF-κB p65 was enhanced in LPS-exposed FGL2^+/+^ pups at P1 and was strikingly inhibited when FGL2 was deleted (**Figure 5B**). *In vitro*, the activation of PI3K/NF-κB pathway was also increased in LPS-treated BMECs and further inhibited by FGL2 deletion (**Figures 5C–E**). These data suggested the PI3K/NF-κB pathway could be the molecular mechanism underlying FGL2-regulated oxidative stress.

To confirm the role of PI3K/NF-κB pathway in FGL2-regulated oxidative stress in BMECs, we firstly pretreated FGL2^+/+^ BMECs with a specific PI3K pathway inhibitor, LY294002, before LPS exposure. Similar to the findings in FGL2-deficient cells, the LPS-induced expressions of iNOS and NOX2, as well as intracellular ROS and NO levels were all downregulated by PI3K inhibitor (**Figures 5F–H**). We further overexpressed PI3K in FGL2^+/+^ and FGL2^-/-^ BMECs by lentivirus. PI3K overexpression had an increasing trend on the expressions of NOX2 and iNOS in PBS-treated FGL2^+/+^ BMECs, and these effects were enhanced by LPS (**Figure S6**). More importantly, the inhibitory effects of FGL2 depletion on NOX2 and iNOS expressions were both blocked by PI3K overexpression in LPS-treated FGL2^-/-^ BMECs (**Figure 5I**). These findings strengthened the regulatory role of PI3K in FGL2-mediated oxidative stress. Additionally, the LPS-induced activation of NF-κB was inhibited in PI3K-inhibited BMECs (**Figure 5J**), but re-activated when PI3K was overexpressed in FGL2^-/-^ BMECs (**Figure 5I**). These results indicated NF-κB was the downstream of PI3K in our experimental systems. Collectively, all of these results strongly supported that PI3K/NF-κB were the key factors in FGL2-mediated endothelial oxidative stress.

## 4 Discussion

It is well recognized that maternal health during pregnancy has a significant impact on offspring outcomes (5). Maternal inflammation during gestation disturbs the processes of fetal neurodevelopment and further causes prenatal brain damage, which ultimately contributes to the short-term and long-term neurological disorders in offspring (1, 3, 5, 37). Thus, unveiling the pathogenesis of maternal inflammation-induced prenatal brain damage is critical for developing new therapeutic approaches. In the present study, we demonstrated the BBB function was impaired in neonatal mice after maternal LPS exposure and highlighted the protective role of FGL2 deficiency in maternal inflammation-induced BBB damage. Furthermore, we uncovered PI3K/NF-κB mediated endothelial oxidative stress was the key pathway involved in FGL2-regulated BBB damage. These results indicated that FGL2 and PI3K/NF-κB pathway could be potential therapeutic targets for maternal inflammation-induced brain damage. To our knowledge, this is the first to report the role of FGL2 in mediating BBB disruption and refine the mechanisms involved.

Blood-brain barrier acts as a protective gatekeeper to maintain cerebral homeostasis in the developing brain (38). The BBB begins to form around E11.5 in mice, is initially functional at E15.5 and continues to mature over a brief period after birth (38, 39). Previous study has indicated during the process of barriergenesis, fetal BBB is vulnerable and could be disrupted rapidly after maternal inflammation exposure (14, 15). Our study subsequently indicated the damage of BBB in neonatal mice exposed to maternal LPS in the maturation period of BBB at E17. Therefore, our results support and extend the previous studies that the immature BBB is susceptible to the prenatal inflammation throughout the periods of formation and maturation. TJs, the basic structure of BBB, is widely reported to be disrupted during inflammation (11). We observed a remarkable reduction of representative TJ proteins in dysfunctional neonatal BBB after maternal LPS exposure. Interestingly, although the latest study from CD-1 mice suggested the disruption of fetal BBB formation after maternal Poly(I:C) exposure, the immunostaining densities of Claudin-5 and ZO-1 remained unchanged in malfunctional fetal BBB (14). LPS and Poly(I:C) models are the most commonly used models to study maternal inflammation, and mimic the infection of bacteria and viruses respectively. LPS is a component of the outer membrane of Gram-negative bacteria and activates Toll-like receptor-4 (TLR4) pathway, whereas Poly(I:C) is a synthetic analogue of the viral double-stranded RNA which activates TLR3 pathway. Similar to our results, previous study indicated LPS seemed more easily to induce TJ reduction than Poly(I:C) in the immature brain due to the activation of different TLRs (40). Thus, the inconsistent results of TJs may result from the differences in inflammatory processes. It further suggested the complexity of BBB damage upon maternal inflammation. Precise investigations and comparisons in different conditions are still needed in the future.

FGL2 has been reported to regulate inflammatory processes by activation of macrophage (17, 41). Whereas, the specific role of FGL2 in neuroinflammation is not completely understood. Using the FGL2-deficient mice, our group firstly reported that FGL2 deficiency markedly alleviated the inflammation response and white matter injury in the offspring’ brain exposed to maternal inflammation, probably *via* microglia polarization (19, 42). However, we noticed the protective effect of FGL2 deletion already existed 6 hours after LPS exposure, which was before the change of microglial phenotypes. Interestingly, the impaired fetal BBB integrity has been observed as early as 6 hours after LPS exposure previously (15). It suggested that there could be other key regulating factors involved in the protective effect of FGL2 deficiency, such as BBB. In this study, we illustrated that FGL2 deficiency significantly mitigated the maternal inflammation-induced hyperpermeability of BBB and the declines of TJ proteins, consequently alleviating neuroinflammation in offspring. Collectively, based on our current and previous findings, we propose that FGL2 deficiency alleviates the perinatal neuroinflammation of offspring by targeting BMECs and microglia. However, the animals we used were systemic knockout mice instead of condition knockout. In order to refine the regulatory effect of FGL2 on BBB, we took advantage of primary BMECs and combined the results of both *in-vivo* and *in-vitro* experiments to strengthen our conclusions. Nevertheless, the specific role of endothelial FGL2 in neuroinflammation needs to be further investigated in condition FGL2 knockout animals. Moreover, sFGL2 has been reported to inhibit pro-inflammatory T helper type 17 (Th17) cell response, while IL-17a, a signature cytokine secreted by Th17 cells, has been proven to promote neuroinflammation and affect neurological phenotypes of offspring (3, 43). Thus, sFGL2 in maternal and fetal circulation may participate in suppressing the pro-inflammatory response during maternal inflammation. Although we used heterozygous dams to partly reserve its level in circulation, it could not exclude the effect of sFGL2 decline on normal immune response. The specific role of two different forms of FGL2 in the process of maternal inflammation-induced brain damage is worth further investigation.

As is well known, microglial cells are the key players in neuroinflammation and also play a critical role in regulating BBB function (10). It is not exactly clear about the link between microglia and BMECs as well as its regulatory effect on BBB properties. However, there is evidence that shows the polarization states of microglia are the decisive factor, wherein M1 microglia contribute to BBB dysfunction and M2 microglia play a protective role in BBB disruption (10). Interestingly, FGL2 deficiency has been shown to reduce M1 polarization and increase M2 polarization of microglia during maternal inflammation in our previous study (19). Thus, FGL2-mediated microglia activation may also contribute to BBB injury during maternal inflammation. Moreover, based on our previous and present results, FGL2 knockout dynamically affected the cytokine levels in fetal brain in distinct time course of maternal inflammation, facilitating the early increase of IL-10 and the later inhibition of TNF-α (19). IL-10 is an important non-microglial cytokine to prevent pathological microglial hyperactivation, while microglia-derived TNF-α can reactivate microglia by a positive feedback mechanism (10, 44). Thus, FGL2-mediated dynamic regulation of cytokines could participate in microglia activation and directly or indirectly lead to BBB dysfunction. In spite of the undefined process of cytokine regulation above, these data implied a complicated FGL2-involved interaction network between microglia and BMECs during maternal inflammation, which needs additional *in-vivo* study using condition FGL2 knockout animals and *in-vitro* experiment by co-culture system.

Emerging evidence indicates TLRs is a putative pathway linking maternal inflammation and fetal brain damage, while less is known about its role in fetal BBB damage (45). TLR4, which is primarily expressed in microglia but also expressed in brain endothelial cells, is the well-reported toll-like receptor to regulate the BBB integrity as well as mediate M1 microglia polarization (45). The activation of TLR4 pathway on these two cells has been shown to contribute to BBB leakage *in vitro (* 46, 47). Meanwhile, intracerebral injection of TLR4 inhibitor decreased the hyperpermeability of BBB in adult mice exposed to intracerebral hemorrhage, while the opposite result was observed after TLR4 overexpression (48). More importantly, the change of BBB function by TLR4 alteration in this research was parallel to its change by FGL2, emphasizing the potential role of TLR4-FGL2 interaction in regulating BBB function (48). In fact, TLR4-FGL2 interaction has been proven to cause the activation of peripheral macrophage by LPS stimulation (17). As the specific ligand of TLR4, intrauterine administration of LPS is capable of accessing the entire feto‐placental unit to reach the fetus (49). Meanwhile, TLR4 antagonist has been proven to alleviate cytokine eruption in fetal brain after maternal LPS exposure (50). Therefore, the potential effect of LPS-TLR4-FGL2 axis on microglial activation or BBB dysfunction, especially during maternal inflammation, is worthy of further study.

As reported, FGL2 has essential physiological functions to several organs, such as sperm maturation in epididymis and smooth muscle contraction in heart (51). However, its physiological role in brain is seldom reported. The expression of FGL2 in brain, including cerebral cortex, is markedly lower than it in peripheral organs, and there is low or null expression of FGL2 in normal neuron cells, neuroglial cells and endothelial cells (52). FGL2 knockout in two strains of mice did not affect the brain weight as well as the brain-to-body weight ratio of offspring during the perinatal period (19). Meanwhile, FGL2 knockdown or overexpression had no effect on the neurological function of control adult mice, whereas FGL2 knockdown could alleviate neurological deficits by intracerebral hemorrhage (48). Moreover, based on our results of NAF extravasation and TJ protein expression in neonatal mice, endogenous FGL2 had no significant effect on normal BBB formation or function, whereas inducible FGL2 by maternal inflammation dramatically participated in BBB disruption. Interestingly, latest research reported that FGL2 overexpression and knockdown had no obvious effect on BBB permeability in control adult mice, but pathological inducible FGL2 remarkably caused BBB hyperpermeability (48). These data collectively proposed that endogenous FGL2 might manipulate BBB function and CNS homeostasis mainly under pathological states due to its abnormal expression.

Oxidative stress, an inevitable component of inflammation, is a crucial mechanism of BBB damage (31, 34, 53). Maternal inflammation during pregnancy has been found to cause oxidative stress in fetal brain (15, 54). Excessive products in oxidative stress, such as ROS, NO, and subsequently peroxynitrite formation, can cause TJs disruption and cytotoxic effects, eventually resulting in BBB injury (34). In the present study, we further confirmed the elevated products of oxidative stress in neonatal brain accompanied by BBB damage. More importantly, FGL2 deficiency remarkably reduced the production of ROS and NO in neonatal brain and in endothelial cells. NOX2-derived ROS and iNOS-derived NO are known to be the key factors of BBB impairment (34). In our study, the endothelial expressions of both two proteins were downregulated by FGL2 deletion. All of these data supported that FGL2 played a vital role in regulating endothelial oxidative stress during maternal inflammation. In addition, PI3K and NF-κB have been well-recognized in endothelial oxidative stress (32, 36), but this is the first to report that FGL2 is involved in this process. Through inhibitor and overexpression experiments, we finally strengthened the critical role of PI3K/NF-κB pathway in the process of FGL2-mediated endothelial oxidative stress.

Even though researchers have generally agreed that the fetal period of brain development is the pivotal time window to prevent later-in-life behavior disorders. There is still a lack of effective treatments in offspring, as well as other FGL2-related neuroinflammatory diseases such as ischemic-reperfusion injury and intracerebral hemorrhage (20, 21). BBB impairment and inflammatory responses are two crucial mechanisms involved in neuroinflammation-related cerebral damage. While, FGL2 deficiency not only alleviates the microglia-mediated inflammation but also shows robust protection against BBB damage. Therefore, FGL2 is not only the potential therapeutic target for clinical treatment of maternal inflammation-induced fetal brain damage, but also an universal target for other neuroinflammatory diseases.

**Figure 6:**
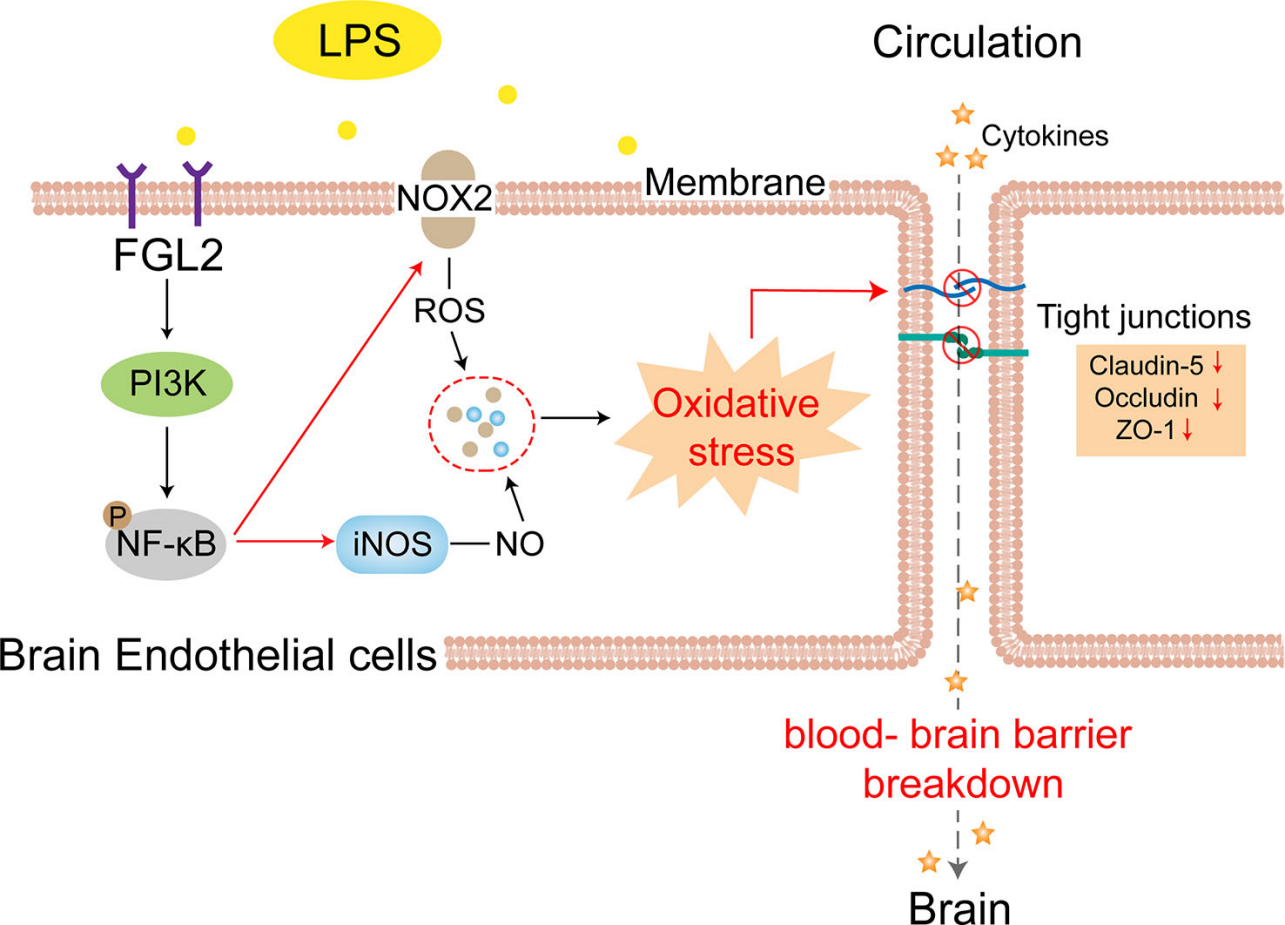
FGL2 deficiency attenuated blood-brain barrier disruption in maternal inflammation-exposed offspring. FGL2 deficiency downregulates the maternal inflammation-induced expressions of NOX2 and iNOS in BMECs *via* inhibiting PI3K/NF-κB pathway, which decreases the accumulation of ROS and NO. The reduced oxidative stress subsequently alleviates the declines of tight junction proteins (Claudin-5, Occludin, and ZO-1), thereby restoring permeability of blood-brain barrier and alleviating neuroinflammation in maternal inflammation-exposed offspring.

In conclusion, we emphasized the importance of BBB dysfunction in the maternal inflammation-exposed developing brain and further uncovered a new mechanism underlying. We found that FGL2 deficiency alleviated blood-brain barrier damage by blocking PI3K/NF-κB-mediated endothelial oxidative stress in maternal inflammation-exposed neonatal brain (**Figure 6**). Our findings strongly suggested that FGL2 could be the potential target for the treatment of prenatal brain damage as well as other neuroinflammatory diseases.

## Data availability statement

The original contributions presented in the study are included in the article/**Supplementary Material**. Further inquiries can be directed to the corresponding authors.

## Ethics statement

The animal study was reviewed and approved by Tongji Hospital Animal Care and Use Committee (number: TJH-201901018).

## Author contributions

LH and DZ designed the research, analyzed the data, and prepared the manuscript. YX and YY performed the animal experiments and analyzed the data. QL, JZ, and SL performed the cell experiments and collected the data. QN provided technical assistance and experimental suggestions. XL and CZ were the corresponding authors. CZ contributed to the conception of the research and prepared the manuscript. XL supervised the research and corrected the manuscript. All authors contributed to the article and approved the submitted version.
