# Supplementary material for: FGL2 deficiency alleviates maternal inflammation-induced blood-brain barrier damage by blocking PI3K/NF-κB mediated endothelial oxidative stress
Source: Front Immunol. 2023 Mar 27;14:1157027. doi: 10.3389/fimmu.2023.1157027 (PMC10083319; doi:10.3389/fimmu.2023.1157027)
Supplement: Supplementary file 1 [file DataSheet_1.docx]

Supplementary Material

FGL2 deficiency alleviates maternal inflammation-induced blood-brain barrier damage by blocking PI3K/NF-κB mediated endothelial oxidative stress

# Supplementary Figures, 2-4

Figure S1. The characterization of gestational maternal inflammation.

Figure S2. The characterization of BMECs.

Figure S3. The cerebral expression of FGL2 was absent in FGL2^-/-^ mice.

Figure S4. FGL2 deficiency rescued the loss of body weight and brain weight in pups.

Figure S5. The placental inflammation of FGL2^+/-^ dams and the cerebral FGL2 expression of FGL2^+/+^ pups were elevated after LPS exposure.

Figure S6. PI3K/NF-κB pathway regulated the endothelial oxidative stress in FGL2^+/+^ BMECs.

# Supplemental Table, 5-6

Table S1: The primary antibodies used for western blotting

Table S2: Primer sequences used for RT-qPCR analysis


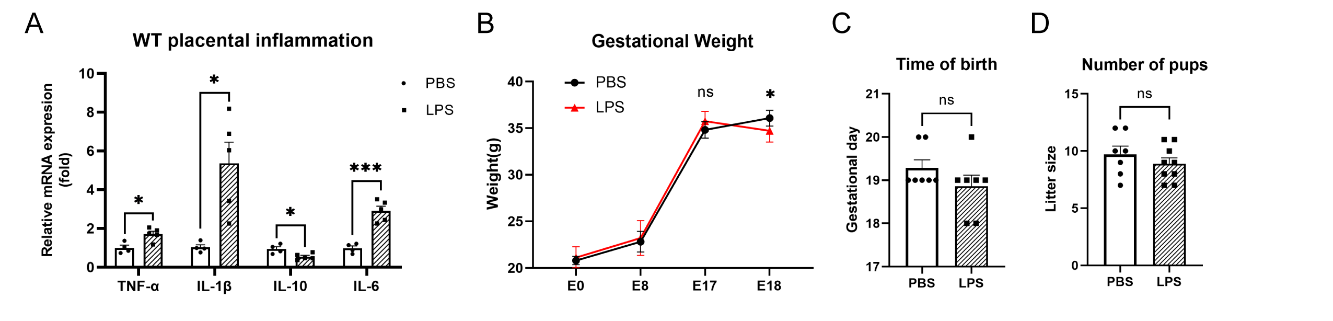


**Figure S1. The characterization of gestational** **maternal inflammation.** (A) The mRNA levels of *TNF-α*, *IL-1β*, *IL-10*, and *IL-6* in the placenta of WT mice (n = 4:5). (B) The maternal weight changes of dams during pregnancy (n = 9). (C) The time of birth in PBS and LPS groups (n = 7). (D) The litter size in PBS and LPS groups (n =7:9). The data was expressed as mean±SEM and was the representative of at least three independent experiments. Statistical differences were assessed by unpaired two-tailed Student’s t test for two groups. ns *P*>0.05, * *P*<0.05, ** *P*<0.01, *** *P*<0.001.

**
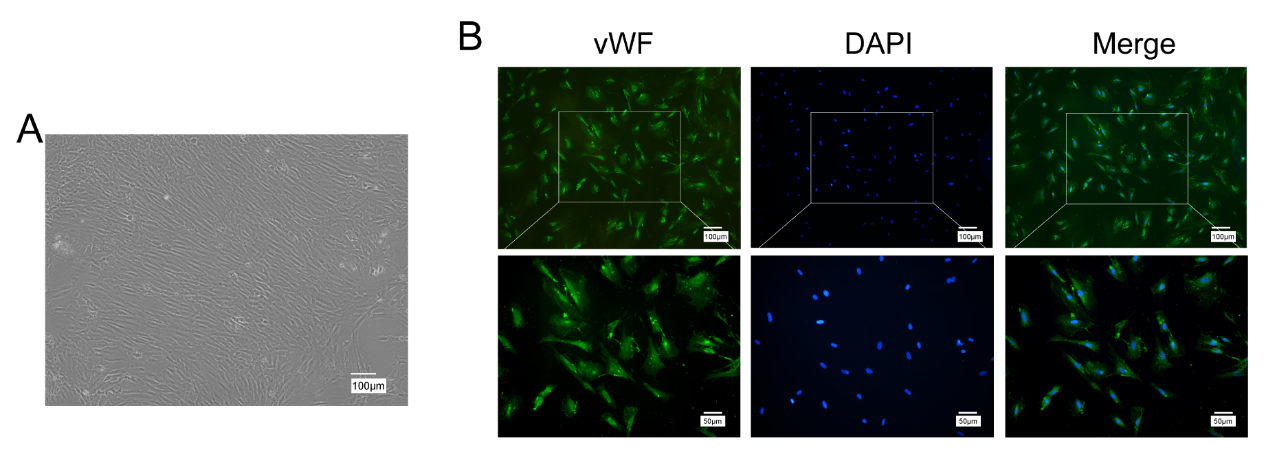
**

**Figure S2. The characterization of BMECs.** (A) Morphology of primary BMECs under the microscope. Scale bar= 100 μm. (B) Primary BMECs expressed vWF by immunofluorescence staining. Representative images were shown. Scale bar is 100 μm for the upper panel and 50 μm for the lower panel.


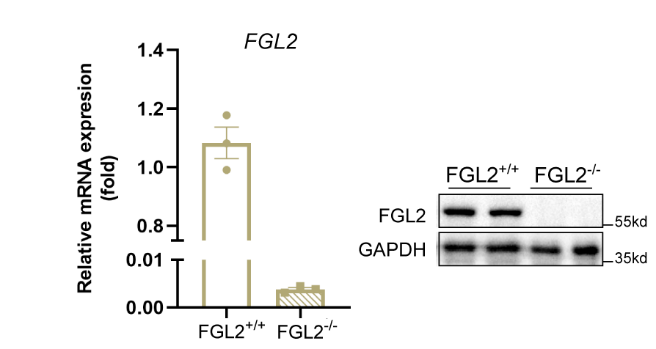


**Figure S3. The cerebral expression of FGL2 was absent in FGL2^-/-^ mice.** The cerebral mRNA and protein levels of FGL2 in mice. The data was expressed as mean±SEM and was the representative of at least three independent experiments. Statistical differences were assessed by unpaired two-tailed Student’s t test for two groups.


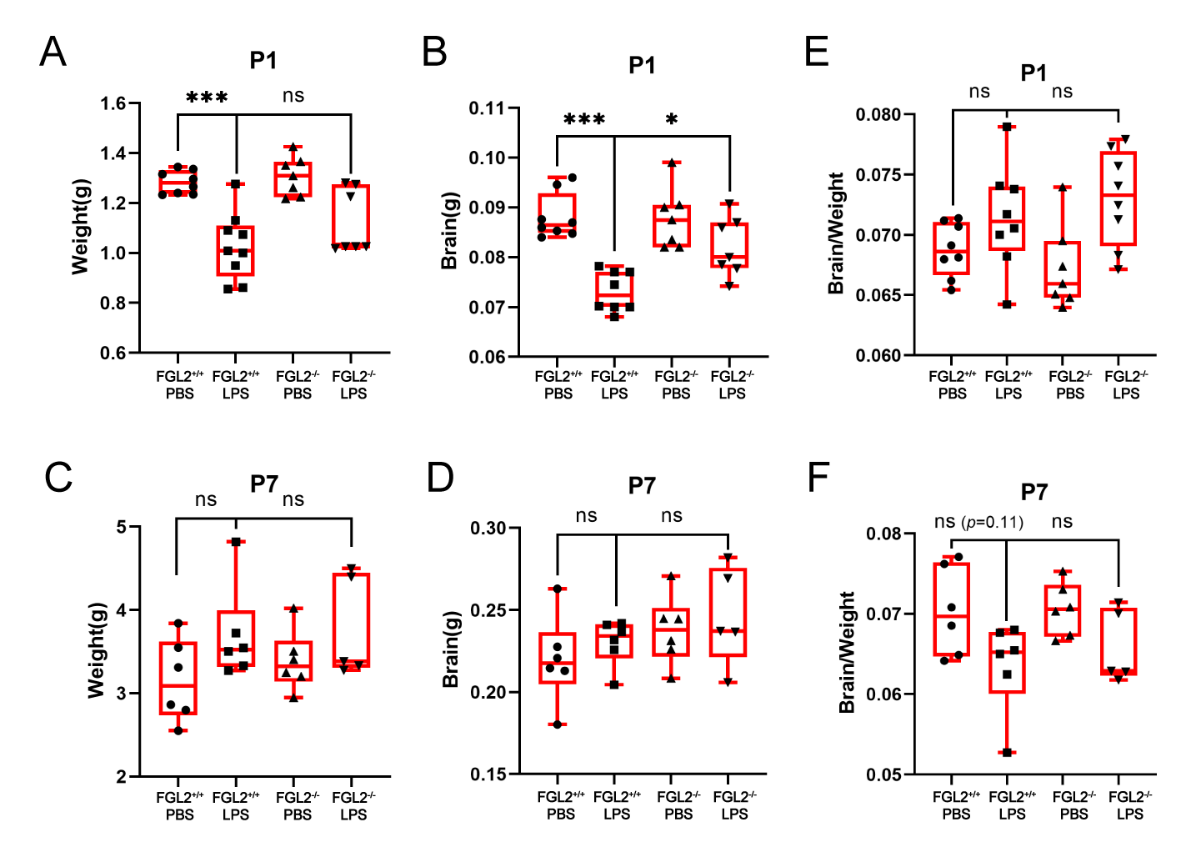


**Figure S4. FGL2 deficiency rescued the loss of body weight and brain weight in pups.** (A) The body weight of pups at P1 (n = 8:9:7:7). (B) The brain weight of pups at P1 (n = 8:8:7:7). (C) The body weight of pups at P7 (n = 6:6:6:5). (D) The brain weight of pups at P7 (n = 6:6:6:5). (E) The ratio of brain weight to body weight in pups at P1 (n = 8:8:7:8). (F) The ratio of brain weight to body weight in pups at P7 (n = 6:6:6:5). The data was expressed as mean±SEM and was the representative of at least three independent experiments. Statistical differences were assessed by one-way ANOVA with Tukey’s post-hoc test for multiple groups. ns *P*>0.05, * *P*<0.05, ** *P*<0.01, *** *P*<0.001.


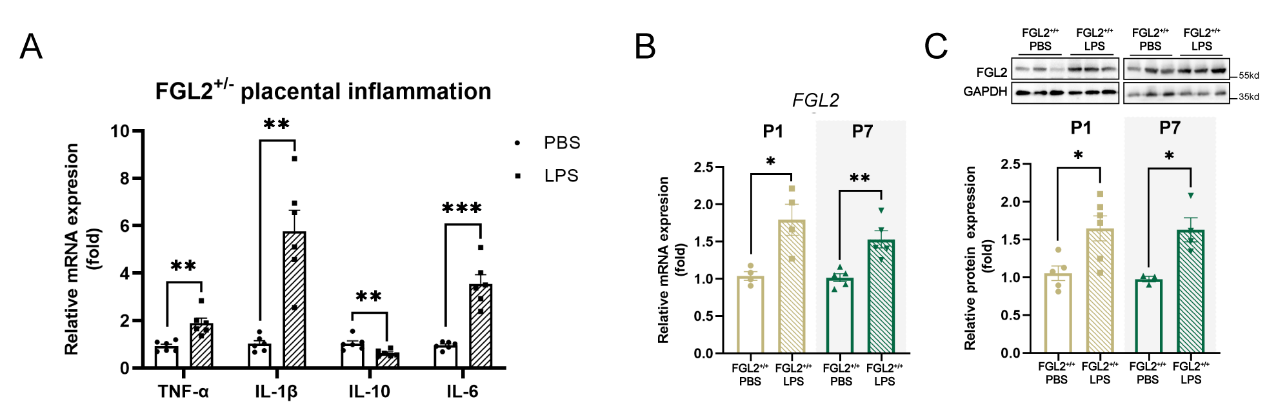


**Figure S5. The placental inflammation of FGL2^+/-^ dams and the cerebral FGL2 expression of FGL2^+/+^ pups were elevated after LPS exposure.** (A) The mRNA levels of *TNF-α*, *IL-1β*, *IL-10*, and *IL-6* in the placenta of FGL2*^+/-^* dams (n = 6). (B) The cerebral mRNA levels of FGL2 in FGL2^+/+^ pups at P1 and P7 (n = 4:4:5:5). (C) The protein expressions of FGL2 in FGL2^+/+^ pups at P1 and P7 (n = 5:6:3:4). The data was expressed as mean±SEM and was the representative of at least three independent experiments. Statistical differences were assessed by unpaired two-tailed Student’s t test for two groups. ns *P*>0.05, * *P*<0.05, ** *P*<0.01, *** *P*<0.001.


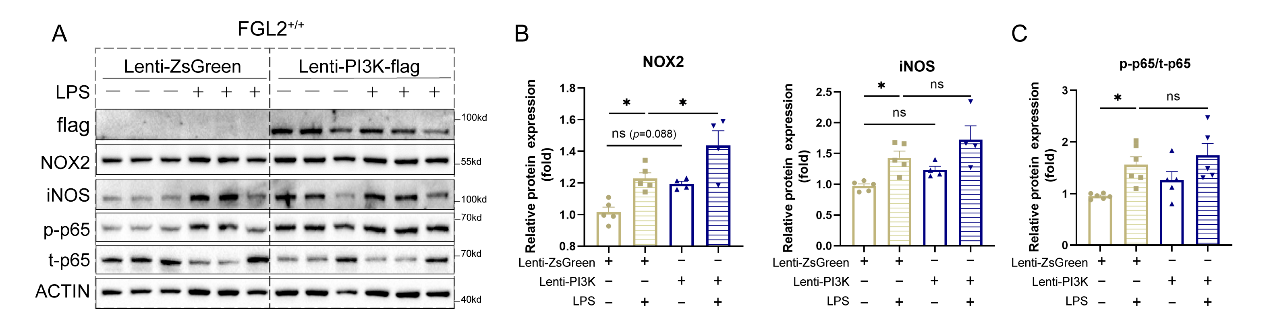


**Figure S6. PI3K/NF-κB pathway regulated the endothelial oxidative stress in FGL2**^+/+^ **BMECs.** (A) Representative western blots. (B) The protein expressions of NOX2 and iNOS in FGL2^+/+^ BMECs (n = 5:5:4:4). (C) The protein expressions of p-p65 and t-p65 in FGL2^+/+^ BMECs (n = 6:6:5:5). FGL2^+/+^ BMECs were infected by Lenti-PI3K-flag or Lenti-ZsGreen before treatment of LPS (5μg/ml) or PBS for 24h. The data was expressed as mean±SEM and was the representative of at least three independent experiments. Statistical differences were assessed by one-way ANOVA with Tukey’s post-hoc test for multiple groups. ns *P*>0.05, * *P*<0.05, ** *P*<0.01, *** *P*<0.001.

**Table S1: The primary antibodies used for western blotting**

| Antigen | Company | Catalog no. | Concentration |
| --- | --- | --- | --- |
| GAPDH | Proteintach | 10494-1-AP | 1:5000 |
| ACTIN | Abclonal | AC026 | 1:100000 |
| FGL2 | Abnova | H00010875-M01 | 1:3000 |
| NOX2 | Abclonal | A19701 | 1:1000 |
| iNOS | Proteintach | 18985-1-AP | 1:1000 |
| ZO-1 | Thermo Fisher Scientific | 61-7300 | 1:2000 |
| Claudin-5 | Thermo Fisher Scientific | 34-1600 | 1:1000 |
| Occludin | Abclonal | A2601 | 1:2000 |
| PI3K p85α | Proteintach | 60225-1-Ig | 1:1000 |
| phospho-NF-κB p65 | Cell Signaling Technology | 3039 | 1:1000 |
| NF-κB p65 | Cell Signaling Technology | 8242 | 1:1000 |
| flag | Cell Signaling Technology | 8146 | 1:1000 |

**Table S2: Primer sequences used for RT-qPCR analysis**

| Target mRNA | Sequence |
| --- | --- |
| Fgl2 | Forward:5’-CAGTCACAGCCGGTTCAACAT-3’ |
|  | Reverse:5’-GTAGACCTCAAAGCTGCTGTTTC-3’ |
| Gapdh | Forward:5’-CAAAATGGTGAAGGTCGGTGTG-3’ |
|  | Reverse:5’-TGATGTTAGTGGGGTCTCGCTC-3’ |
| Pik3r1 | Forward:5’-GGAGCAGCAACCGAAACAAA -3’ |
|  | Reverse:5’-ACTTCGCCGTCTACCACTAC -3’ |
| TNF-α | Forward:5’-CAGGCGGTGCCTATGTCTC-3’ |
|  | Reverse:5’-CGATCACCCCGAAGTTCAGTAG-3’ |
| IL-6 | Forward:5’-TAGTCCTTCCTACCCCAATTTCC-3’ |
|  | Reverse:5’-TTGGTCCTTAGCCACTCCTTC-3’ |
| IL-1β | Forward:5’-ATGCCACCTTTTGACAGTGATG-3’ |
|  | Reverse:5’-TGTGCTGCTGCGAGATTTGA-3’ |
| IL-10 | Forward:5’-GCTCTTACTGACTGGCATGAG-3’ |
|  | Reverse:5’-CGCAGCTCTAGGAGCATGTG-3’ |
| ZO-1 | Forward:5’-ACCACCAACCCGAGAAGAC-3’ |
|  | Reverse:5’-CAGGAGTCATGGACGCACA-3’ |
| Claudin-5 | Forward:5’-ACGGGAGGAGCGCTTTAC-3’  Reverse:5’-GTTGGCGAACCAGCAGAG-3’ |
| Occludin | Forward:5’-AGTACATGGCTGCTGCTGAT-3’ |
|  | Reverse:5’-CCGCAACTGGCATCTCTCTA-3’ |
